# Point of Care Ultrasound (POCUS) Applications Taught Within Canadian Internal Medicine Residency Programs: Results of a National Survey

**DOI:** 10.24908/pocusj.v10i01.18220

**Published:** 2025-04-15

**Authors:** Ryan Marinovich, Michael G. R. Beyaert, Steven J. Montague, Irene W. Y. Ma, Luke A. Devine

**Affiliations:** 1 Queen's University, Division of General Internal Medicine, Department of Medicine, Kingston, ON, CAN; 2 Department of Medicine, University of Toronto, Division of General Internal Medicine, Mt. Sinai Hospital, Toronto, ON, CAN; 3 Department of Medicine, Division of General Internal Medicine, Cumming School of Medicine, University of Calgary, Calgary, Alberta, CAN

**Keywords:** POCUS, education, internal medicine, Canadian, national survey

## Abstract

**Background:**

Point of care ultrasound (POCUS) is an important tool for bedside diagnostics and procedures within internal medicine. In 2017 the Canadian Internal Medicine Ultrasound (CIMUS) group provided recommendations for applications to teach POCUS to internal medicine trainees. The way that training programs have implemented these recommendations has not been assessed. We aim to assess POCUS applications taught within internal medicine training programs, five years after the CIMUS group's recommendations.

**Methods:**

An anonymous survey was distributed in 2022 to POCUS leads at Canadian internal medicine residency and general internal medicine subspecialty training programs. This voluntary survey assessed which POCUS applications were being taught to trainees.

**Results:**

A total of 17 responses for the core training program and 12 responses for the subspecialty training program were collected. There was widespread uptake of the CIMUS-recommended POCUS applications within core and subspecialty level training. The compliance was higher for procedures than diagnostics. Many applications recommended at the subspecialty level were being incorporated into the core training program curriculum. Many applications that were not commented on by the CIMUS group were also incorporated into POCUS curricula.

**Conclusions:**

POCUS education within Canadian internal medicine residency programs are largely in sync with the CIMUS recommendations published in 2017. Many programs have expanded their training beyond the CIMUS group's recommendations.

## Introduction

Increasingly, point of care ultrasound (POCUS) in internal medicine is being recognized as effective in both assisting with bedside diagnostics and with guiding procedures [1–5]. POCUS education is increasingly implemented within internal medicine internationally [6]. Within Canada, the accrediting organization (the Royal College of Physicians and Surgeons of Canada) requires all internal medicine trainees to be competent for multiple procedures where POCUS has become standard of care (e.g., thoracentesis, paracentesis, central venous access). However, there are no accreditation standards that explicitly require POCUS training for diagnostic applications [7]. Due to the relative novelty of using POCUS for diagnostic purposes, there are no standardized POCUS curricula or mandated educational objectives and experiences across the country in internal medicine. As a result, despite internal medicine residents consistently reporting POCUS as important and applicable to their future practice, they continue to report a lack of confidence in their own POCUS abilities [8,9]. In 2011, Ailon et al [10]. surveyed internal medicine division heads and program directors at each of the 17 internal medicine training programs in Canada. They found significant heterogeneity in POCUS education received by residents. At the time, POCUS was an emerging field within internal medicine, without broad uptake and minimal infrastructure in place to support its clinical use among internists. In 2017, the Canadian Internal Medicine Ultrasound (CIMUS) group prepared consensus-based recommendations for internal medicine programs. These recommendations described which POCUS applications should be taught in both core internal medicine training (postgraduate year (PGY) 1-3) as well as within general internal medicine subspecialty training (PGY 4-5) [11]. The CIMUS group subsequently provided recommendations regarding standardized educational indicators [12] which gave guidance for developing high-quality, useful POCUS curricula across the country.

There is currently no mandatory national POCUS curriculum in Canada. Instead, each internal medicine program has been encouraged to develop their own POCUS curriculum. Significant barriers have been identified to expanding POCUS use and the implementation of POCUS curricula, such as lack of training amongst clinical faculty, lack of ultrasound devices and lack of direct supervision during training [13]. Having acknowledged these barriers, we sought to characterize the state of POCUS education within Canada five years after the CIMUS group's initial curricular recommendations. To that end we undertook a national survey of the 17 internal medicine and the 16 general internal medicine subspeciality training programs within Canada to characterize the current state of POCUS applications being taught across the country.

## Methods

We developed a survey (see supplemental material) based on the survey initially completed by Ailon et al. in 2011 to determine which POCUS applications are being taught to medical trainees at the respondent's training program. Specifically, the national survey outlined in Appendix C and reported in Table 6 of Ailon et al. was used as a framework for our survey [10]. The CIMUS-recommended POCUS applications from 2017 were added to the survey. The first draft was then distributed to the authors (IM, LD, SM) for expert feedback to ensure adequate breadth and clarity of language. Based on their feedback and a literature search of POCUS applications in internal medicine, a final survey was produced. This survey separated POCUS applications into the diagnostic domain and procedural-guidance domain. All common internal medicine applications were included, along with a free text box so respondents could add local applications not included in the survey.

The survey was distributed to the POCUS lead at each training program across Canada in February 2022. Responses were received electronically from February 2022 through to November 2022. POCUS leads were identified by their division heads/chairs. The survey was completed by the individual overseeing the POCUS education for the core internal medicine and the general internal medicine subspecialty programs. The individual could be the same for both programs. When there was no POCUS program lead, the survey was distributed to the program director for both the core internal medicine and general internal medicine subspecialty training programs.

All 17 core internal medicine training sites and all 16 general internal medicine subspecialty training sites had an opportunity to contribute data to this study. To maintain anonymity and to not bias responses, no identifying data was collected about respondents or their programs. This survey was voluntary, and no incentives were given.

Ethics approval was granted by the University of Toronto Human Research Ethics Board (approval number :40806). The survey was distributed electronically via Survey Monkey (Survey Monkey Inc. San Mateo, California, USA; www.surveymonkey.com)

Data was compiled and analyzed using descriptive statistics in Microsoft Excel.

## Results

A total of 18 individuals completed the anonymous survey. Six were specific to their core internal medicine training program (PGY 1-3), and one was for the general internal medicine subspecialty program (PGY 4-5) at their site. The remainder of respondents (n=11) answered for both programs. This resulted in 17 responses for the core internal medicine training programs and 12 responses for the general internal medicine subspecialty training program.

The CIMUS-recommended POCUS applications were incorporated in the majority (mean 83 ± 15%) of core internal medicine training programs (Table 1). Procedural POCUS applications were taught at most sites. All sites reported teaching thoracentesis and central line placement with POCUS, and 16 of the 17 sites reported teaching paracentesis with POCUS. Diagnostic POCUS applications (e.g., assessment of pleural effusions, A & B lines, inferior vena cava, and abdominal ascites) also had good uptake; however, less so than procedural applications. Between 65% and 76% of sites reported implementation of various diagnostic applications in their POCUS curriculum in comparison to 94-100% of sites reporting implementation of procedural applications.

**Table 1.** Implementation of Canadian Internal Medicine Ultrasound (CIMUS)-recommended point of care ultrasound (POCUS) applications at the core internal medicine programs (PGY 1-3).

POCUS Application	Proportion	N
Thoracentesis	100%	17/17
Central Line Placement	100%	17/17
Paracentesis	94%	16/17
Pleural effusion	76%	13/17
A and B lines	76%	13/17
IVC	71%	12/17
Ascites	65%	11/17

Results are displayed as a percentage of all respondents answering for that training level who reported teaching the listed application at their training program, as well as the absolute values (N). A total of 17 responses for the core training level were collected. A and B lines refer to lung ultrasound assessment, IVC refers to inferior vena cava assessment.

The CIMUS-recommended POCUS applications were also incorporated in the majority (mean 67 ± 18%) of the general internal medicine subspecialty training programs (Table 2). The least frequently taught POCUS application was assessment of jugular venous pressure and knee effusions. Many procedural applications (e.g., arterial line placement, peripheral intravenous insertion, arterial blood gas sampling and knee arthrocentesis) also had less uptake.

**Table 2.** Implementation of Canadian Internal Medicine Ultrasound (CIMUS)-recommended point of care ultrasound (POCUS) applications at the general internal medicine subspecialty programs (PGY 4-5).

Application	Proportion	N
IVC	83%	10/12
Pleural effusion	83%	10/12
A and B lines	83%	10/12
Thoracentesis	83%	10/12
Paracentesis	83%	10/12
Pneumothorax	83%	10/12
Pericardial Effusion	83%	10/12
Ascites	75%	9/12
Central Line	75%	9/12
LV Systolic Function	75%	9/12
Consolidation	67%	8/12
RV strain	67%	8/12
Knee Arthrocentesis	50%	6/12
Art Line	50%	6/12
JVP	42%	5/12
Knee Effusion	42%	5/12
Peripheral IV	42%	5/12
ABG	33%	4/12

Results are displayed as a percentage of all respondents answering for that training level who reported teaching and utilization of the listed application at their centre as well as absolute values (N). A total of 12 responses were collected for the subspecialty training level. IVC refers to inferior vena cava size and collapse, LV: left ventricle systolic function, RV: right ventricle, JVP: jugular venous pressure, ABG: arterial blood gas, Art line: arterial line insertion.

Respondents also reported that additional applications were taught in their training programs (Table 3). In general, more applications were incorporated into subspecialty training curricula than in the core training program. Also, many applications recommended for the subspecialty level were incorporated into the POCUS curricula for core internal medicine training.

**Table 3.** Additional point of care ultrasound (POCUS) applications taught at core and subspecialty training programs.

		Core Programs		Subspecialty Programs	
	Application	Proportion	N/17	Proportion	N
Subspecialty Applications	Pericardial Effusion	71%	12	83%	10/12
	Pneumothorax	71%	12	83%	10/12
	LV Systolic Function	65%	11	75%	9/12
	RV strain	53%	9	67%	8/12
	Consolidation	53%	9	67%	8/12
	Arterial line Placement	53%	9	50%	6/12
	ABG	41%	7	33%	4/12
	Knee Arthrocentesis	18%	3	50%	6/12
	Peripheral intravenous	29%	5	42%	5/12
	Hydronephrosis	24%	4	50%	6/12
	Lumbar Puncture	18%	3	42%	5/12
	Bladder Volume	18%	3	25%	3/12
	AAA	12%	2	33%	4/12
	DVT	12%	2	12%	1/12
	Abscess	12%	2	33%	4/12
	LV Diastolic Function	12%	2	33%	4/12

Results are displayed as a percentage of all respondents answering for that training level who reported teaching the listed application at their center as well as absolute values (N). A total of 17 responses were collected for the core training program and 12 responses were collected for subspecialty training program. Gray section represents applications in which the Canadian Internal Medicine Ultrasound (CIMUS) recommendation was to incorporate at the subspecialty level. LV: left ventricle, RV: right ventricle, ABG: arterial blood gas, AAA: abdominal aortic aneurysm, DVT: Deep vein thrombosis.

## Discussion

This is the first national survey to assess which POCUS applications are taught in all internal medicine and general internal medicine residency training programs in Canada. These results serve as a pulse-check on the state of POCUS education within the field of internal medicine in Canada. They come five years after the CIMUS group provided its initial recommendations[11,12] to help guide internal medicine POCUS curricula. These data provide an important update and contrast to the 2011 nationwide survey by Ailon et al. [10]. Our results demonstrate substantial growth in Canadian internal medicine POCUS education over the last 11 years.

Of note is the near-ubiquitous teaching of ultrasound guidance for procedural applications. In 2011, Ailon et al. found that only 56% and 67% of respondents reported teaching ultrasound guidance in paracentesis and thoracentesis respectively, compared to the current 100 % and 94%. This suggests that ultrasound guidance for these procedures has indeed become the standard of care. Diagnostic applications have also risen sharply. Only a handful of respondents reported using POCUS to assess pleural effusions and abdominal ascites in 2011 (22% and 33%, respectively) [10], whereas now the majority of respondents indicate residents at their center are taught these applications (76% for pleural effusions, 65% for ascites).

The CIMUS-recommended POCUS applications are now incorporated into the core internal medicine curriculum at the majority of training programs (Table 1). Furthermore, many POCUS applications are being taught beyond the initial CIMUS recommendations. This includes both additional applications, as well as teaching subspecialty applications to core trainees (Table 3). There remains a minority of programs that do not cover certain applications. Interpreting the applications taught in the general internal medicine subspecialty programs require some consideration. Some general internal medicine subspecialty programs did not teach core internal medicine-level POCUS applications. However, this may be because these applications were believed to be taught during core training and were not considered a good use of curricular time (Table 2).

Many respondents indicated that POCUS applications are being taught beyond the CIMUS group's recommendations, both at the core and subspecialty programs (Table 3). Examples include assessments for hydronephrosis and the use of POCUS in lumbar punctures. This shows that the POCUS educational landscape is quickly evolving, with a high degree of heterogeneity as each program builds its POCUS curriculum. As this landscape changes, the initial CIMUS recommendations from 2017 may need to be revisited. However, the quality of POCUS teaching for each application remains unknown. For any application, we do not know how it is taught or assessed, and to what level of exposure/competency/mastery. Further assessment of the quality of POCUS curricula and competency of applications being taught would be beneficial.

Our study is limited in that it was conducted on a voluntary basis. The surveys did not track any respondent's location, so regional biases could not be determined. While we had 17 respondents for the 17 Canadian core internal medicine programs, we only had 12 respondents for the 16 Canadian general internal medicine subspecialty programs. The four missing respondents may bias our results, as it represents 25% of all general internal medicine programs. It is unclear if the lack of response is due to busy clinical schedules, or if it is related to lack of a POCUS curricula. If the latter were true, our results may overestimate the degree of POCUS applications taught within Canadian general internal medicine subspecialty training program by up to 33% (assuming all non-responders taught zero POCUS applications). The definition of POCUS expert is also abstract and was defined purely on whoever the general internal medicine division chair named. Nonetheless, our methodology is in keeping with several other published national surveys on POCUS [7, 10, 11].

While our study is based upon the work reported by Ailon et al., our survey asks somewhat different questions. For example, while Ailon et al. reported on the clinical use of a particular application at a training site, our survey asks if that application is being taught in their training curriculum. This may confound direct comparisons between these two surveys. While we hope that POCUS training curricula would closely align with local use, as teaching depends on local expertise, we cannot be certain this is the case. Lastly, a limitation of all surveys is that they rely on self-reported data. We have not verified the accuracy of the claims reported in our survey through other means. There may also be a tendency to over/under-report based on personal biases of respondents. We suspect the anonymous nature of the survey limited these biases.

## Conclusion

POCUS education within Canadian internal medicine training programs have grown immensely since the last national survey 11 years ago. While the majority of training programs have incorporated the CIMUS group's curricular recommendations, teaching of recommended applications is not yet universal across all training programs. Many applications beyond the scope of the initial recommendations are also being taught. This is a quickly evolving field of medical education, which will benefit from ongoing development and updates on guidelines and research.


