# Supplementary material for: Point of Care Ultrasound (POCUS) Applications Taught Within Canadian Internal Medicine Residency Programs: Results of a National Survey
Source: POCUS J. 2025 Apr 15;10(1):27–31. doi: 10.24908/pocusj.v10i01.18220 (PMC12057461; doi:10.24908/pocusj.v10i01.18220)
Supplement: Supplementary file 1 [file pocusj-10-01-18220-s001.pdf]

POCUS Applications Survey

Program Declaration

This survey asks questions about the Point of Care Ultrasound (POCUS) applications taught to trainees at your institution, at both the Core Internal Medicine level (PGY 1-3) and at higher levels (PGY 4-5). Some respondents may be involved with only the PGY 1-3 or the PGY 4-5 level, while others may be involved with both.

Throughout the survey, please select responses that reflect the POCUS curriculum in which you are involved. If you are involved in the POCUS curricula for both the PGY 1-3 and the PGY 4-5 programs, you may respond on behalf of both.

1. Please indicate whether you are responding to this survey on behalf of the **PGY 1-3 program**, the **PGY 4-5 program**, or **both**.

- ◯ PGY 1-3
- ◯ PGY 4-5
- ◯ Both

POCUS Applications Survey

Procedural Point of Care Ultrasound Applications

Definitions:
Ultrasound Guidance – Use of ultrasound prior to, or while, performing the procedure to identify an appropriate location to insert a needle.

2. **Ultrasound Guided Procedures**. Which of the following procedures are taught using **Ultrasound Guidance** to trainees in your program? If a procedure is taught using POCUS in your curriculum, please select all that apply to indicate whether it is taught at the **PGY 1-3 and/or PGY 4-5 level.**

	PGY 1-3	PGY 4-5
Thoracentesis	☐	☐
Paracentesis	☐	☐
Lumbar Puncture	☐	☐
Central Venous Catheter (CVC) Insertion	☐	☐
Peripheral Intravenous Catheter (PIV) Insertion	☐	☐
Arterial Blood Gas (ABG) Sampling	☐	☐
Arterial Line Insertion	☐	☐
Knee Arthrocentesis	☐	☐
Abscess Drainage	☐	☐

3. List any other procedures that are taught using ultrasound to trainees in your program. Please specify **type of procedure**, whether it is taught using **Ultrasound Guidance**, and whether it is taught at the **PGY 1-3 and/or the PGY 4-5 level.**

POCUS Applications Survey

Diagnostic Point of Care Ultrasound Applications

Which of the following diagnostic applications are included in your program's Point of Care Ultrasound curriculum? If a diagnostic application is taught in your curriculum, please select all that apply to indicate whether it is taught at the PGY 1-3 and/or the PGY 4-5 level.

4. Cardiac Applications

	PGY 1-3	PGY 4-5
Pericardial effusion	☐	☐
Left ventricular systolic function	☐	☐
Left ventricular diastolic function	☐	☐
Right ventricular function/strain	☐	☐
Valvular disease	☐	☐

5. Lung Applications

	PGY 1-3	PGY 4-5
Pleural effusion	☐	☐
Pneumothorax	☐	☐
Interstitial syndrome (i.e. A- and B-lines)	☐	☐
Consolidation	☐	☐

6. Abdominal Applications

	PGY 1-3	PGY 4-5
Free fluid/ascites	☐	☐
Bowel obstruction	☐	☐
Gallbladder	☐	☐
Biliary system	☐	☐

7. Genitourinary Applications

	PGY 1-3	PGY 4-5
Hydronephrosis	☐	☐
Bladder volume	☐	☐

8. Vascular Applications

	PGY 1-3	PGY 4-5
Abdominal aortic aneurysm (AAA)	☐	☐
Inferior vena cava (IVC)	☐	☐
Jugular venous pressure (JVP)	☐	☐
Deep vein thrombosis (DVT) - Lower Limb	☐	☐
Deep vein thrombosis (DVT) - Upper limb	☐	☐

9. Skin and Soft Tissue Applications

	PGY 1-3	PGY 4-5
Abscess	☐	☐
Cobblestoning	☐	☐
Necrotizing fasciitis	☐	☐
Joint effusion - Knee	☐	☐
Joint effusion - Other joint(s) (please specify which joint(s) in the Comment Box at the bottom of this page)	☐	☐
Synovitis - Knee	☐	☐
Synovitis - Other joint(s) (please specify which joint(s) in the Comment Box at the bottom of this page)	☐	☐

10. List any other diagnostic POCUS applications that are taught to trainees in your program. Please **specify the application** and whether it is taught at the **PGY 1-3 and/or the PGY 4-5 level**.
